# Highly Conductive 3D Segregated Graphene Architecture in Polypropylene Composite with Efficient EMI Shielding

**DOI:** 10.3390/polym9120662

**Published:** 2017-12-02

**Authors:** Fakhr E. Alam, Jinhong Yu, Dianyu Shen, Wen Dai, He Li, Xiaoliang Zeng, Yagang Yao, Shiyu Du, Nan Jiang, Cheng-Te Lin

**Affiliations:** 1 Key Laboratory of Marine Materials and Related Technologies, Zhejiang Key Laboratory of Marine Materials and Protective Technologies, Ningbo Institute of Materials Technology and Engineering (NIMTE), Chinese Academy of Sciences, Ningbo 315201, China; alam@nimte.ac.cn (F.E.A.); ShenDianYu@yeah.net (D.S.); daiwen@nimte.ac.cn (W.D.); lihe@nimte.ac.cn (H.L.); 2 University of Chinese Academy of Sciences, 19 A Yuquan Rd., Shijingshan District, Beijing 100049, China; 3 Shenzhen Institutes of Advanced Technology, Chinese Academy of Sciences, Shenzhen 518055, China; xl.zeng@siat.ac.cn; 4 Division of Advanced Nanomaterials, Key Laboratory of Nanodevices and Applications, CAS Center for Excellence in Nanoscience, Suzhou Institute of Nano-tech and Nano-bionics, Chinese Academy of Sciences, University of Chinese Academy of Sciences, Suzhou 215123, China; ygyao2013@sinano.ac.cn; 5 Division of Functional Materials and Nanodevices, Ningbo Institute of Materials Technology and Engineering, Chinese Academy of Sciences, Ningbo 315201, China; dushiyu@nimte.ac.cn

**Keywords:** conductive polymer composites, electrical properties, thermal properties, thermoplastics resin

## Abstract

The extensive use of electronic equipment in modern life causes potential electromagnetic pollution harmful to human health. Therefore, it is of great significance to enhance the electrical conductivity of polymers, which are widely used in electronic components, to screen out electromagnetic waves. The fabrication of graphene/polymer composites has attracted much attention in recent years due to the excellent electrical properties of graphene. However, the uniform distribution of graphene nanoplatelets (GNPs) in a non-polar polymer matrix like polypropylene (PP) still remains a challenge, resulting in the limited improvement of electrical conductivity of PP-based composites achieved to date. Here, we propose a single-step approach to prepare GNPs/PP composites embedded with a segregated architecture of GNPs by coating PP particles with GNPs, followed by hot-pressing. As a result, the electrical conductivity of 10 wt % GNPs-loaded composites reaches 10.86 S·cm^−1^, which is ≈7 times higher than that of the composites made by the melt-blending process. Accordingly, a high electromagnetic interference shielding effectiveness (EMI SE) of 19.3 dB can be achieved. Our method is green, low-cost, and scalable to develop 3D GNPs architecture in a polymer matrix, providing a versatile composite material suitable for use in electronics, aerospace, and automotive industries.

## 1. Introduction

The production of high electrically conductive polymer composites has attracted attention due to their applications in many fields, including electrostatic and electromagnetic interference shielding (EMI), conductive thin films, a flexible electrode in light emitting diodes, and many others [1,2,3,4,5,6]. Electromagnetic interference shielding effectiveness (EMI SE) strongly depends on the electrical conductivity of the shielding materials [7]. Generally, conductive fillers are introduced into the polymer to fabricate conductive polymer composites [8]. The electrical conductivity of polymer composites depends mainly on intrinsic conductivity, aspect ratio, and content of the fillers [9,10,11]. Graphene is a promising filler compared to other conductive nanofillers, which endows polymers with a variety of properties such as electrical conductivity, electromagnetic interference shielding, as well as mechanical and thermal reinforcement [12,13]. Compared to other carbon nanomaterials, graphene has received attention due to its exceptional young modulus (1 TPa), surface area (2630 m^2^·g^−1^) and electrical conductivity (6000 S·cm^−1^) [14]. However, it is still a challenge to incorporate GNPs in the polymer matrix with well-defined microstructure, good interfacial strength, and uniform dispersion [12,15].

To bestow the excellent properties of GNPs to the polymer matrix is to realize GNPs-based polymer composite with a homogenous dispersion of graphene in the resultant composite. Conventionally, melt-blending, solution blending, and in-situ polymerization techniques have been used to fabricate graphene-based polymer composites [16]. However, with the composites prepared by the above-mentioned methods, the exceptional properties of graphene are not fully exploited. The reason is the poor dispersion of graphene in the polymer due to the strong interactions between the graphene sheets, which limits the improvements of the properties in the polymer composites. It is especially difficult to achieve a homogeneous dispersion of graphene in a nonpolar polymer like polypropylene (PP), due to the large polarity difference and low interaction energy [17]. To overcome this problem, chemical functionalization of the graphene surface has been commonly employed [18]. Though, the functionalization of graphene can improve the dispersability but damage the electronic conjugation of graphene sheets, resulting in the degradation of electrical conductivity of the composite [12]. Additionally, the various types of defects originating from chemical functionalization restrict the application of conductive graphene composite [13]. Therefore, it is necessary to develop an easy and general approach to prepare conductive graphene/polymer composite.

The recent development of 3D GNPs network inside the polymer composite represents a potential solution, for avoiding graphene agglomeration and providing a uniform dispersion of graphene in the polymer matrix [17]. The polymer composite with conductive 3D interconnected network demonstrates excellent EMI SE [19,20]. Graphene foam, sponge, and aerogel have been successfully obtained with the 3D graphene network [21,22,23]. However, the two-step process to develop graphene monolith first followed by infusion with the polymer is complicated, time-consuming, and difficult to scale-up. The construction of the 3D segregated graphene network in the polymer provides a new strategy to develop highly conductive polymer composites. The 3D segregated graphene network significantly reduces the contact resistance between GNPs. In such a 3D network, GNPs reside at the interface between the polymers. However, these conductive networks suffer from drawbacks such as inferior mechanical properties, poor interfacial interaction between the filler and matrix, and an imperfect segregated graphene network [20]. Therefore, there is a strong demand to have a simple and versatile approach to fabricate a compact 3D segregated GNPs/PP composite with high electrical conductivity and good mechanical properties.

Here we report a simple and scalable approach to prepare hot-pressed PP composites embedded with segregated architecture by a one-step fabrication, which is time and cost-efficient compared to the conventional process. At the same loading of graphene, the electrical conductivity of PP composites with the segregated graphene architecture is ≈7 times higher than those of melt blending samples with randomly distributed graphene [24,25]. At 10 wt % GNPs content, the electrical conductivity of GNPs/PP composites with the formation of segregated GNPs architecture is 10.86 S·cm^−1^. The EMI SE of the GNPs/PP at 10 wt % loading is 19.3 dB. The EMI SE is close to the target value (20 dB), required for commercial application [26]. The segregated architecture leads to enhancement in thermal stability, storage modulus, and glass transition temperature of the composite as compared to neat PP.

## 2. Materials and Methods

### 2.1. Materials

Graphene nanoplatelets (GNPs) prepared by intercalation and exfoliation of graphite were supplied by Ningbo Morsh Technology Co., Ltd., Ningbo, China. PP powder was purchased from Samsung, Seoul, Korea. The molecular weight of purchased PP is 42.02 g·mol^−1^. Dehydrated ethanol was obtained from Sinopharm Chemical Reagent Co., Ltd., Shanghai, China. All the materials were used without further purification.

### 2.2. Sample Preparation

The as-received GNPs were washed with dehydrated ethanol in order to remove the adsorbed moisture. Before mixing with PP, the calculated amount of GNPs and PP were sonicated for 5 min in ethanol and then mixed together under magnetic stirring for 40 min. The ethanol was removed by filtration to obtain GNPs/PP powder, followed by drying in an oven at 80 °C for 2 h. A stainless steel mold with 30 × 30 mm in length × width and 1 mm thickness was used to contain GNPs/PP powder, and then GNPs/PP composites were fabricated by uniaxial hot-pressing for 10 min under 10 MPa at the melting point of PP (160 °C). The average thickness of the samples after hot-pressing is around 0.90 mm. Our process is simple, scalable and time consuming compared to other methods.

### 2.3. Characterizations

The lateral size and thickness of GNPs were examined by an optical microscope (OM) (Leica, Wetzlar, Germany) and atomic force microscope (AFM) (Dimension 3100, Veeco, Plainview, NY, USA), respectively. The microstructure of GNPs was studied by transmission electron microscopy (TEM) (JEM-2100F, JEOL, Tokyo, Japan). The morphology of GNPs and GNPs/PP composites was observed by field emission scanning electron microscopy (FE-SEM) (Quanta FEG 250, FEI, Waltham, MA, USA). The quality of GNPs was investigated by Raman Spectroscopy (Renishaw plc, Wotton-under-edge, UK) with a laser wavelength of 532 nm, as well as X-ray photoelectron spectroscopy (XPS) (Axis Ultra DLD, Kratos Analytical, Kyoto, Japan). The electrical conductivity was calculated by using the Hall Effect measurement system (Hall 8800, Super solutions & Services Co., Ltd., Hsinchu, Taiwan). The EMI shielding of the GNPs/PP composite was measured using a network analyser, Agilent N5242A PNA-X, (Agilent, Santa Clara, CA, USA) , USA. The dimension of the samples was 22.5 × 10 mm (length × width) to measure the EMI SE. The EMI SE experiment was carried out in the frequency range 8–12 GHz. Thermogravimetric analyses (TGA) was characterized by a TGA 209 F3 (NETZSCH, Hamburg, Germany). The samples were close to 10 mg and all the measurements were performed under nitrogen (20 mL/min) and air (20 mL/min) atmosphere. The measurements were carried out in the range of 30 to 800 °C at a heating rate of 20 °C /min. Dynamic mechanical analysis (DMA) was performed on a DMA Q800 dynamic mechanical analyzer (TA Instruments, New Castle, DE, USA), operating in tension mode at an oscillation frequency of 1 Hz. The melting point of the polymers was evaluated by using differential scanning calorimeter (DSC) (PYRIS Diamond™, PerkinElmer, Waltham, MA, USA). The measurement was carried out in the range of 50 to 250 °C at a heating rate of 10 °C/min.

## 3. Results

**Figure 1:**
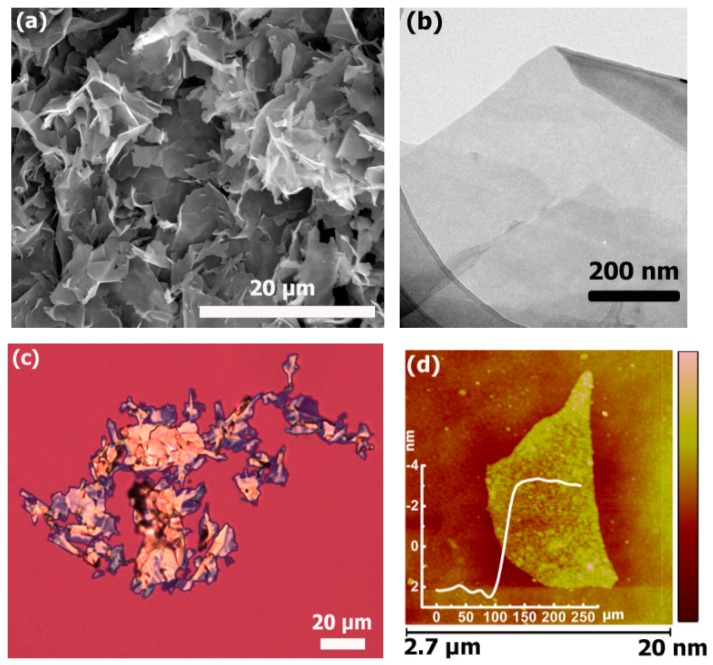
The characterization of GNPs: (**a**) SEM; (**b**) TEM image of individual GNPs; (**c**) OM and (**d**) AFM.

The morphological and structural characterizations of GNPs were performed before preparation of the GNPs/PP composites. The SEM image Figure 1a of GNPs shows a flake-like structure and the laminar configuration can be seen on the surface of the GNPs. A thin sample was observed in the TEM shown in Figure 1b, revealing a smooth surface morphology without cracks or pinholes. Figure 1c,d present the OM and AFM images of GNPs, respectively, which show the polygonal and flat appearance. In Figure 1c, the typical OM image shows the morphology of GNPs deposited on a Si substrate by dip-coating. The color of the GNPs on SiO_2_ (300 nm)/Si substrate is shiny yellow to yellow-green, which is not similar to the observations of a monolayer or several-layer graphene indicating that the GNPs are composed of graphene multilayer [17,27]. The AFM image of individual GNPs is displayed in Figure 1d. The average literal size and average thickness is around 5.4 ± 0.3 μm and 10.6 ± 0.3 nm which we calculated in our previous work [17]. Figure S1a exhibits a typical Raman spectrum of GNPs with a small D-band (at 1353 cm^−1^), a strong, sharp G-band (at 1580 cm^−1^), and a prominent 2D-band (at 2713 cm^−1^). The occurrence of D-band can be attributed to defect introduction during the production process based on intercalation/exfoliation of graphite [28]. The chemical composition of GNPs was investigated by XPS analysis. As a fitting result in Figure S1b, the spectrum of C1s binding energy consists of four components: C=C/C–C (sp2/sp3 bonding, ≈284.8 eV), C–O (hydroxyl group, ≈285.3 eV/285.7), C=O (carbonyl group, ≈288.3/287.4 eV), and π × π* (291.1 eV) [29,30]. It is concluded that GNPs are comprised of 94.0% carbon and 6.0% oxygen-containing functional groups.

**Figure 2:**
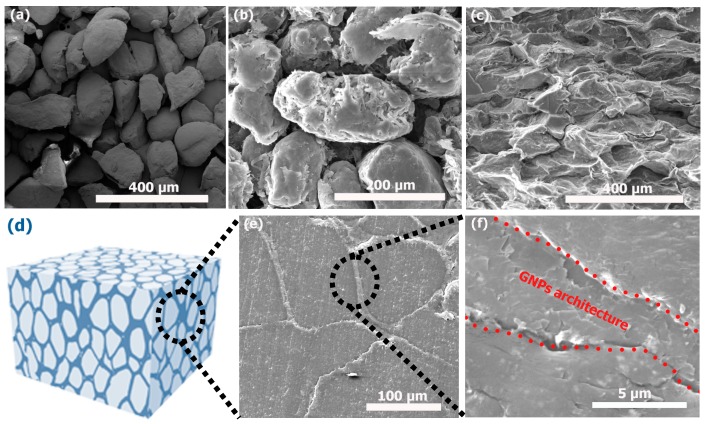
SEM images of (**a**) neat PP (**b**) GNPs-coated PP (**c**) the section view of before polishing hot pressed composite (**d**) scheme of composite (**e**) after polishing the surface (**f**) the magnification of (**e**).

The SEM images of obtained PP and the sample incorporated with GNPs are displayed in Figure 2. Figure 2a displays the SEM image of the PP powder before coating with GNPs. The pristine PP powder presents a rough shape with an irregular contour, and the particle size is in the range of 100–150 µm. The SEM images of the PP powder, after coating with 10 wt % GNPs, are shown in Figure 2b. After deposition, we found that the surface of the powder particles is covered by a thin layer of GNPs and the morphology of PP mostly remains the same. GNPs prefer to be physically attached to the surface of the microparticles due to their relatively strong adhesive interaction, based on the high aspect ratio (>550) and ultrathin nature of GNPs. Figure 2c presents the cross-sectional view of the GNPs/PP composite after hot-pressing. It is observed that the PP powder has been melted partially and integrated due to high pressure (10 MPa) and temperature (160 °C). Figure 2d presents the scheme of the composite obtained after hot-pressing. Figure 2e and f display low and high magnification SEM images of the GNPs/PP composite. As the sample surface was carefully polished, evidence of the formation of segregated graphene architecture within the polymer matrix was revealed. The boundary of PP particles is surrounded by lean strips composed of GNPs, which are connected together to form a continuous segregated architecture throughout the entire composite. From the investigation of the microstructure, we suggest that the PP particles were not fully covered by GNPs, resulting in maintenance of the mechanical strength of the composite [17].

**Figure 3:**
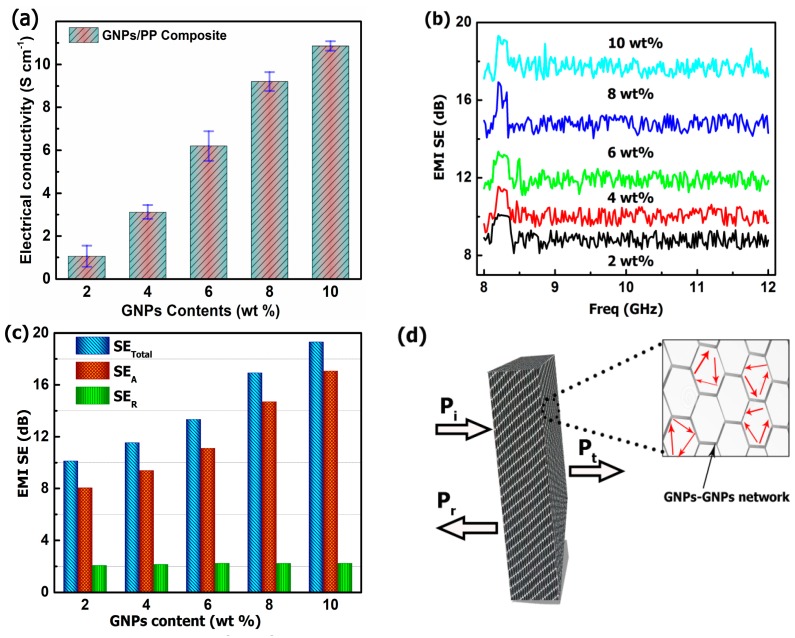
(**a**) Electrical conductivity of the GNPs/PP composite and their comparison with previous work at different loading amounts (**b**) EMI SE of GNPs/PP composite as function of frequency (X-band) (**c**) the comparison of SE_Total_ SE_A_ and SE_R_ at different loading amount of GNPs (**d**) schematic representation of microwave transfer across the composite.

It has been proved that constructing a continuously conductive network in composites is of crucial importance for achieving highly conductive composite at low filler contents. The highly ordered 3D GNPs architecture makes a great contribution to the properties of the composite materials. The influence of segregated architecture on the electrical conductivity of GNPs/PP composite was studied and the conductivity variation is presented in Figure 3a as a function of GNPs loading. The electrical conductivity of the GNPs/PP composite is observed to increase with increasing content of GNPs. The GNPs/PP composite with 2 wt % GNPs exhibits conductivity of 1.06 S·cm^−1^, which is nine orders of magnitude higher than neat PP (1 × 10^−9^ S·cm^−1^) [8]. The highest conductivity of the hot-pressed GNPs/PP composite at 160 °C is 10.86 S·cm^−1^. The outstanding electrical conductivity can be attributed to the formation of the 3D segregated GNPs architecture in the PP matrix. It can be seen in Figure 2c that at 160 °C the PP powder is partially melted and merged together without breaking the 3D GNPs architecture, formed throughout the entire composite. As a result of the formed GNPs architecture, the electrons may be able to hop from a nanosheet to an adjacent one, which leads to the increased conductivity. Furthermore, Table S1 displays the comparison of our result with the previous literature. Based on the same filler content, it is evident that the electrical conductivity of our sample is higher than other approaches adopted to prepare conductive polymer composites. Additionally, the conductivity of the sample is superior to other nanocarbons used to fabricate conductive polymer composites [6,31,32,33,34]. Our conductivity is higher than Wu et al. who follow a complicated process to obtain 3D segregated architecture with a conductivity of 10.24 S·cm^−1^ [1]. The high electrical conductivity of GNPs/PP composite is due to low inter-sheet junction contact resistance, their contacting probability increases, and the majority of GNPs contribute in the building of a continuously conductive network. As a result, our proposed process enables the issue of fabricating highly electrically conductive PP composites to be overcome, including the difficulty of graphene dispersion and the defects occurring in graphene while designing a foam structure [15].

The EMI SE is defined as the logarithm of the ratio of incident power (P_i_) to transmitted power (P_t_) in dB, i.e., SE = −10 log (P_i_/P_t_). In this study, the EMI SE of GNPs/PP composite with an average thickness of 0.90 mm, length 22.5 mm, and width 10 mm was measured in the X-band (8−12 GHz). Figure 3b shows the EMI SE of GNPs/PP composite with various loadings of GNPs as a function of incident frequency. EMI SE shows weak frequency dependence after 8.2 GHz for all samples. The EMI SE almost remains constant as the frequency increases from 8.2–12 GHz. The EMI SE of the composite with 2 wt % GNPs shows variation from 8.1–10 dB over the frequency range of 8–12 GHz. However, the average EMI SE of 4 wt % GNPs-loaded PP composite is 10.07 dB, indicating that ≈ 90% of the electromagnetic radiation is blocked by the shielding material [35]. It is clear from Figure 3b that the EMI SE strongly depends on the content of GNPs in the composites. The EMI SE of GNPs/PP at 10 wt % loading of GNPs varies from 17–19.3 dB over the X-band frequency range. The average EMI SE of the GNPs/PP composite is 17.72 dB with 10 wt % GNPs contents. The increased EMI SE is attributed to the enhanced electrical conductivity of the GNPs/PP composite. Additionally, the creation of a 3D segregated GNPs architecture in the composite shows strong interaction with an electromagnetic wave and leads to the enhancement of EMI SE, due to the formation of an interconnected electrically conductive network.

The interaction of the electromagnetic waves with materials generates effects such as transmission, reflection, and absorption. To further study the EMI SE in GNPs/PP composites, we calculated the total EMI SE (SE_Total_) which is the sum of the shielding effect due to reflection SE_R_ and absorption SE_A_ from the measured scattering parameters (S11 and S21). Figure 3c reveals the dependence of the SE_Total_, SE_R_, and SE_A_ on GNPs loading at a frequency of 8.2 GHz. The SE_A_ and SE_R_ increase with increasing GNPs content but the increase in SE_R_ is negligible as compared to the SE_A_. For example, when the GNPs content is increased from 2–10 wt %, the SE_A_ increased from 8.05–17.06 dB (111%), whereas SE_R_ increased from 2.07–2.24 dB (8.2%), indicating that the contribution to SE_Total_ from absorption and reflection is 88% and 12% respectively. It is evident that absorption dominated the shielding behavior. The SE_Total_ of GNP/PP composite at 8.2 GHz incorporated with 10 wt % GNPs reached 19.3 dB, suggesting that about 99% of the incident radiation is stopped by the material [36]. A minimum SE_Total_ of 20 dB is required for commercial applications. The scheme of Figure 3d presents the mechanism of the EMI SE process. The P_i_ is divided into P_r_, and P_t_. As shown in Figure 3d the entrance of the electromagnetic wave in the composite would be reflected, scattered, and adsorbed by the development of segregated GNPs architecture multiple times, leading to the rapid attenuation of the electromagnetic wave energy. Eventually, most of the incident electromagnetic waves are absorbed and converted to heat before escaping from the composite [31]. Only a small amount of power might be reflected by the sample surface.

**Figure 4:**
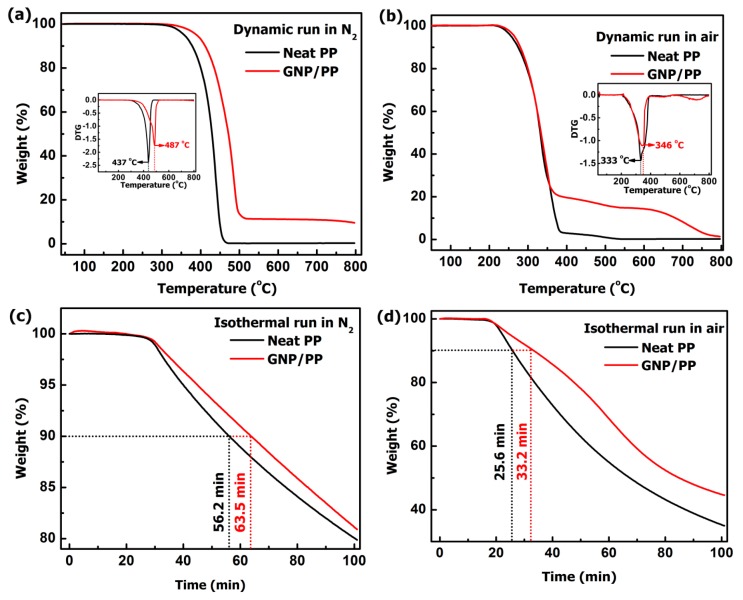
TGA curve of neat PP and 10 wt % GNPs (**a**) dynamic run in N_2_ with differential thermogravimetric analysis (DTG) (**b**) dynamic run in air with DTG (**c**) isothermal run at 350 °C in N_2_ (**d**) isothermal run at 250 °C in air.

TGA was carried out in both nitrogen (N_2_) and air atmosphere to study the thermal stability of PP and GNPs/PP composite, presented in Figure 4a–d. Figure 4a shows the TGA curve of neat PP and GNPs/PP composite in N_2_ with a heating rate of 20 °C/min. N_2_ (20 mL/min) was used as inert gas to prevent unwanted oxidation of samples by removing air from the pyrolysis zone [37]. Figure 4a reveals that the degradation temperature of neat PP and GNPs/PP is 437 and 487 °C respectively. The thermal stability of the GNPs/PP increased by 50 °C upon introduction of 10 wt % GNP compared to neat PP (inset Figure 4a). In the N_2_ environment, the initial weight loss was due to the degradation of PP in the temperature range 350–500 °C. The slow weight loss is due to the barrier effect of GNP lean strip on the surface of PP that is destroyed at higher temperature [38]. Figure 4b presents the thermogram carried out in air environment with a heating rate of 20 °C/min. The GNPs/PP composite shows thermal stability in air as compared to neat PP. The addition of GNPs results in a shift of the peak to a higher temperature (inset Figure 4b). It is revealed from the comparison of Figure 4a and Figure 4b that the degradation of the sample starts at a lower temperature in the air atmosphere. It indicates that the degradation of the neat PP and GNPs/PP composite is enhanced due to oxygen by random scission at the major weight loss stage. Isothermal TGA curves of neat PP and GNPs/PP composite in both N_2_ and air are presented in Figure 4c,d, respectively. The annealing temperature is 350 °C for N_2_ and 250 °C for air environment respectively. It is clear from Figure 4c,d, that the addition of GNPs into the PP matrix retard the thermal degradation of the polymer. GNPs/PP composites took a long time to degrade to 90 wt % compared to neat PP in both N_2_ and air atmosphere. In the case of GNPs/PP composites under N_2_, as shown in Figure 4c, the times for 90 wt % PP and GNPs/PP display 56.2 and 63.5 min, respectively. Similarly, in the air atmosphere, the times for 90 wt % PP and GNPs/PP display 25.6 and 33.2 min, respectively as shown in Figure 4d. These results show that the thermal stability of GNPs/PP composite with 10 wt % GNPs, is increased and the reason is that the addition of filler restricts the movement of polymer chain segments and molecules, leading to an enhanced decomposition temperature [37].

**Figure 5:**
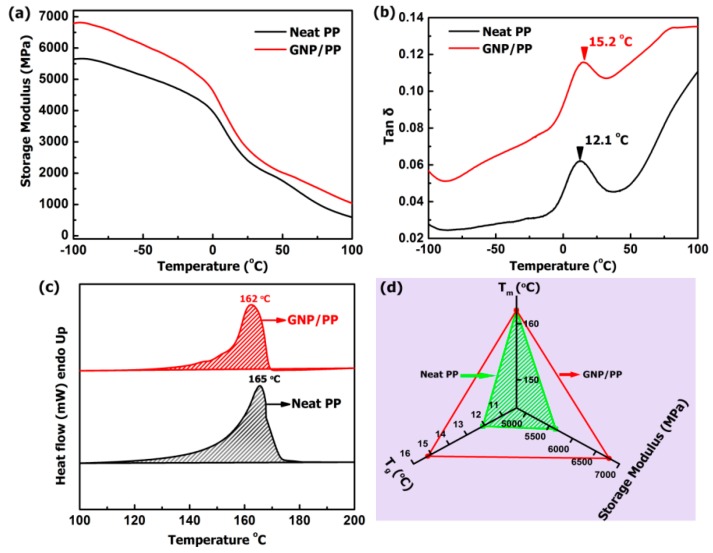
(**a**) Storage modulus (**b**) loss factors (**c**) DSC curves and (**d**) radar chart presentation of three parameters of the PP and GNPs/PP composites.

It is revealed from Figure 2b that the PP particles are not fully covered by the GNPs; this will affect the dynamics of the polymer chain to some extent. Figure 5a,b present the storage modulus and loss factor as a function of temperature. Figure 5a shows that a remarkable enhancement in storage modulus is observed for the GNPs/PP composite. The storage modulus of neat PP is 5639 MPa and that of GNPs/PP composite is 6784 MPa at −100 °C. At 10 wt % loading of GNPs, the storage modulus of the composite increased by 20.3% at −100 °C compared to neat PP. The increase in storage modulus is 15.3% at room temperature (25 °C). This suggests that the segregated GNPs architecture in GNPs/PP composite would have a positive influence on the mechanical properties. The improvement in the storage modulus indicates a strong interaction between matrix chains and GNPs. GNPs have shown maximum surface contact with the PP matrix due to the high aspect ratio of the filler. The storage modulus for neat PP and GNPs/PP decreases with increasing temperature. Figure 5b shows the temperature dependence of the loss factor for the GNPs/PP composite. The loss factor tan δ is defined as the ratio of the loss modulus to the storage modulus, which is very sensitive to solid structural transformation in materials. The tan δ peak value is used to determine the transition temperature (*T*_g_). The *T*_g_, measured at the peak of tan δ, shows an increase of 3.1 °C at 10 wt % GNPs. This is attributed to the reduced chain mobility during dynamic mechanical deformation. Figure 5c shows the DSC thermograms of the neat PP and GNP/PP composite. The melting temperature (*T*_m_) of the neat PP and GNPs/PP composite is 165 and 162 °C, respectively. A slight change is observed in the melting point. Figure 5d presents radar charts that compare three parameters of the neat PP and GNPs/PP. The difference in the volume of the triangular pyramid indicates the improvement in the storage modulus and *T*_g_ of the GNPs/PP composites while the *T*_m_ of the neat PP and GNPs/PP remains almost unchanged.

## 4. Conclusions

In summary, we developed a time- and cost-efficient approach to fabricate GNPs/PP composites with remarkably enhanced electrical conductivity. Our concept is to uniformly coat GNPs on the surface of PP powder by mixing in ethanol first, followed by drying and then hot-pressing. Different from the samples made by the melt-blending or solution-mixing process, a feature of segregated graphene architecture embedded in the PP matrix can be observed in our samples. The in-situ formation of the electrically percolating framework of GNPs during hot-pressing enables significant enhancement of electrical conductivity (10.86 S·cm^−1^) of the resulting composites. At 10 wt % loading of GNPs, the EMI SE of PP composites reaches 19.3 dB. Moreover, we found that the composite exhibits significant enhancement in dynamic mechanical properties and thermal stability compared to neat PP.

## Supplementary Materials

The supplementary materials are available online at www.mdpi.com/2073-4360/9/12/662/s1.
