# Supplementary material for: Highly Conductive 3D Segregated Graphene Architecture in Polypropylene Composite with Efficient EMI Shielding
Source: Polymers (Basel). 2017 Dec 2;9(12):662. doi: 10.3390/polym9120662 (PMC6418531; doi:10.3390/polym9120662)
Supplement: Supplementary file 1 [file polymers-09-00662-s001.docx]

**SUPPORTING INFORMATION**

Highly Conductive 3D Segregated Graphene Architecture in Polypropylene Composite with Efficient EMI Shielding

Fakhr E. Alam ^1,2^, Jinhong Yu ^1*^, Dianyu Shen ^1^, Wen Dai ^1,2^, Nan Jiang ^1*^, Cheng-Te Lin ^1,2*^

*^1^ Key Laboratory of Marine Materials and Related Technologies, Zhejiang Key Laboratory of Marine Materials and Protective Technologies, Ningbo Institute of Materials Technology and Engineering (NIMTE), Chinese Academy of Sciences, Ningbo 315201, China. E-mail:* [*yujinhong@nimte.ac.cn*](mailto:yujinhong@nimte.ac.cn) *(J.H Yu);* [*jiangnan@nimte.ac.cn*](mailto:jiangnan@nimte.ac.cn) *(N. Jiang);* [*linzhengde@nimte.ac.cn*](mailto:linzhengde@nimte.ac.cn) *(C.-T. Lin)*

*^2^ University of Chinese Academy of Sciences, 19 A Yuquan Rd., Shijingshan District, Beijing 100049, China.*

** Correspondence:* [*linzhengde@nimte.ac.cn*](mailto:linzhengde@nimte.ac.cn) *; Tel.: +86 158-6736-2138*

**SUPPLEMENT**


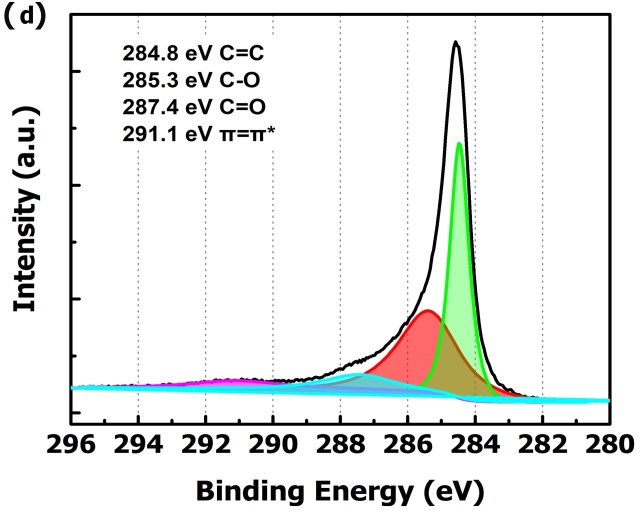

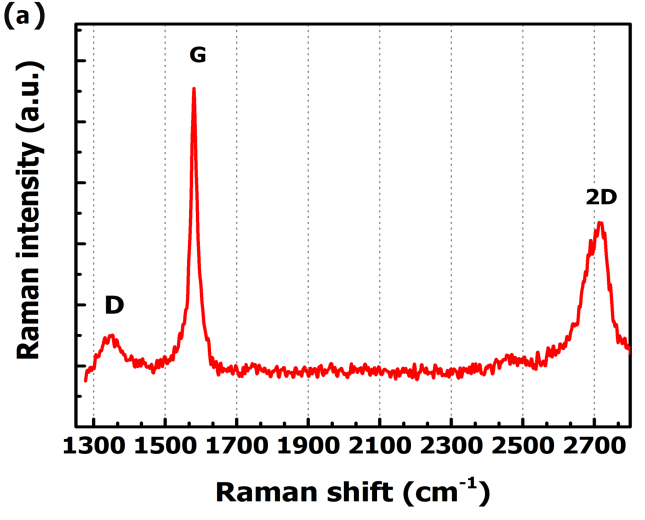


**Figure S1**. (**a**) Raman and (**b**) XPS C1s spectra of GNPs.

**Table S1**. A comparison of the thermal conductivity and TCE of thermoplastic composites made with graphene and white graphene (boron nitride, BN).

| Filler | EC | Content wt% | Matrix | Method | Journal |
| --- | --- | --- | --- | --- | --- |
| Graphene | 2.3 ×10^-2^ S cm^-1^ | 10 | Epoxy | 3D foam Filling | [1] |
| EG | 1.2 × 10^-1^ S ·cm^-1^ | 10 | PEEk | Solution mixing | [2] |
| MWNT | 1 S cm^-1^ | 10 | UHMWPE | Dry mixing | [3] |
| CRGO | 11 ×10^-2^ S cm^-1^ | 10 | PA | Hot pressing | [4] |
| CNT | 3.3* 10^-6^ S cm^-1^ | 10 | ABS | Twin-screw extruder | [5] |
| GO | 0.5 ×10^-2^ S cm^-1^ | 10 | PVA | Ultra sonication and Starring | [6] |
| GSs | 10.24 S cm^-1^ | ≈ 10 | PS | Hot pressing | [7] |
| GNPs | 10.86 S cm^-1^ | 10 | PP | Hot Pressing | This work |

References

1. Ming, P.; Zhang, Y.; Bao, J.; Liu, G.; Li, Z.; Jiang, L.; Cheng, Q. Bioinspired highly electrically conductive graphene–epoxy layered composites. *RSC Adva.* **2015**, *5*, 22283-22288.
2. Goyal, R.K. Cost-efficient high performance polyetheretherketone/expanded graphite nanocomposites with high conductivity for emi shielding application. *Mater. Chem. Phys.* **2013**, *142*, 195-198.
3. Al-Saleh, M.H.; Jawad, S.A.; El Ghanem, H.M. Electrical and dielectric behaviors of dry-mixed cnt/uhmwpe nanocomposites. *High Perfor. Polym.* **2013**, *26*, 205-211.
4. Ma, M.; Zhu, Z.; Wu, B.; Chen, S.; Shi, Y.; Wang, X. Preparation of highly conductive composites with segregated structure based on polyamide-6 and reduced graphene oxide. *Mater. Lett.* **2017**, *190*, 71-74.
5. Jyoti, J.; Basu, S.; Singh, B.P.; Dhakate, S.R. Superior mechanical and electrical properties of multiwall carbon nanotube reinforced acrylonitrile butadiene styrene high performance composites. *Composites Part B: Engin.* **2015**, *83*, 58-65.
6. Yang, J.-H.; Lee, Y.-D. Highly electrically conductive rgo/pva composites with a network dispersive nanostructure. *J. Mater. Chem.* **2012**, *22*, 8512-8517.

7. Wu, C.; Huang, X.; Wang, G.; Lv, L.; Chen, G.; Li, G.; Jiang, P. Highly conductive nanocomposites with three dimensional, compactly interconnected graphene networks via a self‐assembly process. *Adv. Funct. Mater.* **2013**, *23*, 506-513.
